# Interlocked DNA nanostructures controlled by a reversible logic circuit

**DOI:** 10.1038/ncomms5940

**Published:** 2014-09-17

**Authors:** Tao Li, Finn Lohmann, Michael Famulok

**Affiliations:** 1 Chemical Biology and Medicinal Chemistry Unit, Life and Medical Science (LIMES) Institute, University of Bonn, 53121 Bonn, Germany; 2 Center of Advanced European Studies and Research (CAESAR), Ludwig-Erhard-Allee 2, 53175 Bonn, Germany

## Abstract

DNA nanostructures constitute attractive devices for logic computing and nanomechanics. An emerging interest is to integrate these two fields and devise intelligent DNA nanorobots. Here we report a reversible logic circuit built on the programmable assembly of a double-stranded (ds) DNA [3]pseudocatenane that serves as a rigid scaffold to position two separate branched-out head-motifs, a bimolecular i-motif and a G-quadruplex. The G-quadruplex only forms when preceded by the assembly of the i-motif. The formation of the latter, in turn, requires acidic pH and unhindered mobility of the head-motif containing dsDNA nanorings with respect to the central ring to which they are interlocked, triggered by release oligodeoxynucleotides. We employ these features to convert the structural changes into Boolean operations with fluorescence labelling. The nanostructure behaves as a reversible logic circuit consisting of tandem YES and AND gates. Such reversible logic circuits integrated into functional nanodevices may guide future intelligent DNA nanorobots to manipulate cascade reactions in biological systems.

## Abstract (web summary)

DNA is a particularly useful molecule with which to assemble mechanical nanodevices with controllable functions. Here, the authors present a three-membered DNA catenane as a controllable and reversible logic circuit.

## Graphical abstract

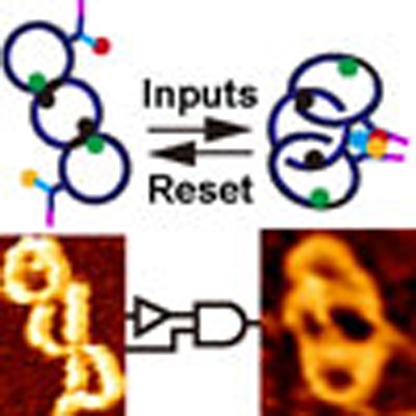

DNA molecules have been extensively used as a highly versatile construction material to build nanomechanical devices such as DNA machines^1^,^2^,^3^,^4^, motors^5^,^6^,^7^,^8^, walkers^9^,^10^,^11^,^12^ and robots^13^,^14^. Moreover, DNA is also useful for assembling molecular computing units including logic gates^15^,^16^,^17^,^18^, circuits^19^,^20^,^21^, calculators^20^,^22^,^23^, and automatons^24^,^25^. Recently, these two fields of application were linked by designing a DNA origami-based nanorobot guided by an aptamer-encoded logic gate^14^, highlighting an emerging interest to integrate DNA-made mechanical nanodevices with molecular computing. An ultimate goal is to construct intelligent DNA nanorobots that carry out complex and programmable tasks *in vivo* or in advanced nanomachinery. However, a huge remaining challenge is the reversibility of integrated DNA-made systems. While most of the known DNA mechanical devices display good reversibility^1^,^2^,^3^,^4^,^5^,^7^,^8^, only very few DNA computing units work repeatedly^21^, illustrating the difficulties to face when devising DNA scaffolds that integrate molecular computing with functional nanodevices.

In DNA architectures like rotaxanes^26^,^27^,^28^,^29^,^30^ or catenanes^31^,^32^,^33^,^34^,^35^,^36^, the interlocked parts can be switched between stalled and mobile states^28^,^29^,^33^,^34^, without falling apart into individual components. Moreover, in contrast to flexible interlocked single-stranded (ss) DNA architectures^33^,^34^,^35^, double-stranded (ds) DNA counterparts are more rigid and easily visualized by atomic force microscopy (AFM)^26^,^27^,^29^. These features are advantageous for Boolean logic-guided operations as they allow precise positioning on a rigid, yet mobile scaffold. Interlocked nanoarchitectures made of dsDNAs thus provide an ideal platform for combining nanomechanical devices with molecular computing.

Here we devise and synthesize a dsDNA [3]pseudocatenane with two branched-out heads as a rigid and stable scaffold. On addition of toehold release oligodeoxynucleotides (ROs), the interlocked and immobile nanocircles comprising the [3]pseudocatenane architecture become mobile. This causes the C-rich regions in the head-motifs to form a bimolecular i-motif structure under acidic conditions. We then employ this interlocked DNA architecture to construct a reversible logic circuit via monitoring its programmable assembly by fluorescence quenching (FQ) and fluorescence resonance energy transfer (FRET). The designed logic circuit is further used to control a DNAzyme nanoswitch built on the difference in robustness between the bimolecular i-motif and G-quadruplex structures on the head-motifs of the interlocked DNA architecture.

## Results

### Design and assembly of the [3]pseudocatenane

**Figure 1:**
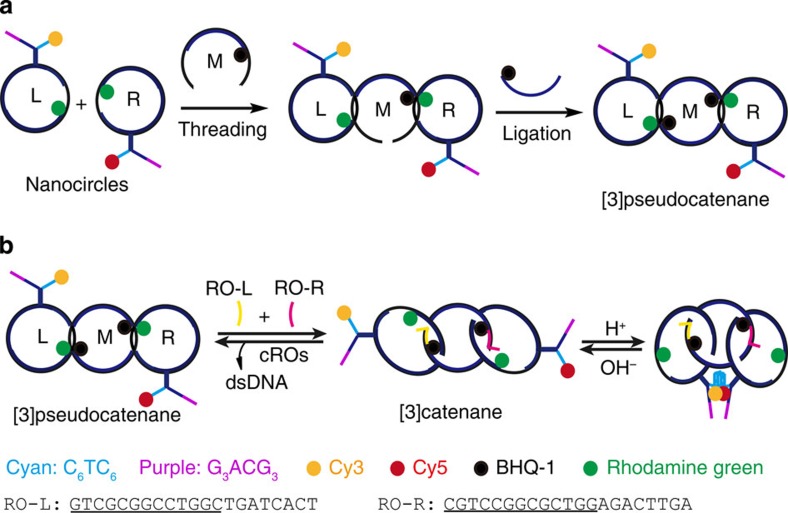
Schematic for the synthesis and programmable assembly of dsDNA [3]pseudocatenane. (**a**) The synthesized [3]pseudocatenane contains C-rich (cyan, C_6_TC_6_) and G-rich (purple, G_3_ACG_3_) branches. The rings L-M and M-R are held together by hybridization in short sections, to form the interlocked structure. (**b**) Addition of RO-L and RO-R (that is, ROs) triggers a structural conversion from [3]pseudocatenane to [3]catenane, where RO-L and RO-R (underlined: the active sequences) are hybridized with two gaps of ring M to displace L and R thereby allowing them to move freely. A bimolecular i-motif (cyan) can then form in the presence of H^+^, resulting in head-motif cyclization in the interlocked system.

**Figure 2:**
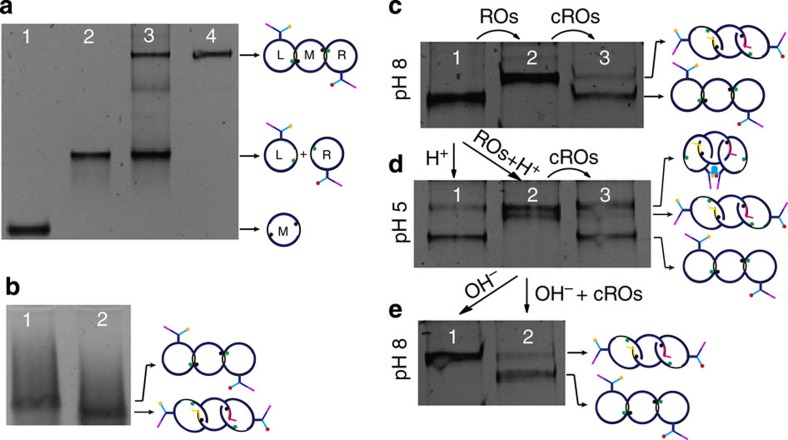
Electrophoretograms of the synthesis and assembly processes of interlocked DNA nanostructures. (**a**) Electrophoretogram of 1 pmol interlocked architecture and each components in 6% native polyacrylamide gel electrophoresis at pH 8. (1) M circle; (2) L and R circles; (3) raw product; (4) pure product. The new band in lane 3 with the slowest mobility corresponds to the formed [3]pseudocatenane. (**b**) Electrophoretogram of 1 pmol assembled [3]catenane in 2.5% agarose gel at pH 8. (1) [3]pseudocatenane; (2) [3]pseudocatenane plus 10 equivalent of ROs. The shift in the mobility of bands originates from the freely moving circles of the [3]catenane (lane 2 versus lane 1). (**c**–**e**) Electrophoretograms of 1 pmol interlocked DNA nanostructures (50 nM, 6% native polyacrylamide gels). (**c**) (1) [3]pseudocatenane at pH 8; (2) 1 plus 10 equivalent of ROs; (3) 2 plus 20 equivalent of cROs. An opposite shift in the mobility of bands is observed. (**d**) (1) [3]pseudocatenane plus H^+^; (2) [3]pseudocatenane plus ROs and H^+^; (3) 2 plus cROs. The two close bands in lane 2 correspond to [3]catenane before and after the cyclization of heads. (**e**) (1) Lane 2 in panel **d** plus OH^−^ (2) lane 2 in panel **d** plus cROs and OH^−^. The addition of OH^-^ results in a disappearance of the slowest band (lane 1), indicating the disruption of i-motif structure at pH 8.

**Figure 3:**
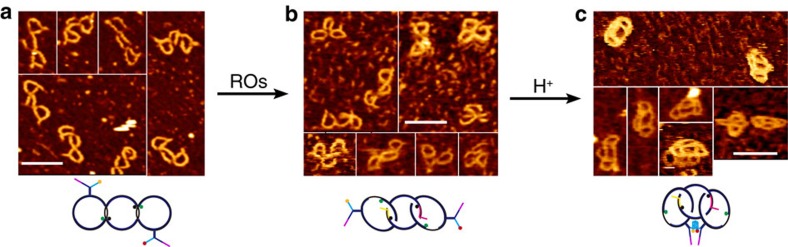
AFM confirmation of the DNA nanoarchitectures. (**a**) AFM image of [3]pseudocatenane at pH 8, with two clearly observed heads. (**b**) AFM image of [3]catenane with ROs at pH 8 in irregular shapes, implying the mobility of circles. (**c**) AFM images of [3]catenane with ROs at pH 5, demonstrating that two heads are linked together. It originates from the formation of bimolecular i-motif structure on the heads. The scale bars represent 50 nm. The whole images corresponding to (**a**–**c**) are provided as Supplementary Figs 2–4.

The synthesized [3]pseudocatenane (Fig. 1a), which is verified by a new and very slowly moving band (Fig. 2a, lane 3) in native polyacrylamide gel electrophoresis, consists of a dsDNA nanocircle with two 13-mer ss-gap regions in the middle (M) to which two dsDNA nanocircles with a 10-mer ss-gap region are interlocked (L and R). Rings L and R also contain a branched-out head-motif bearing one half of a bimolecular i-motif sequence and half of a potential bimolecular G-quadruplex structure. The ss-gaps of the nanocircles L and R are complementary to the gaps in M to which they hybridize so that the branched-out motifs arrange in opposite directions (Fig. 1a). This design ensures that the nanocircles L and R are stably positioned with their two heads held separate, thereby preventing the formation of the bimolecular i-motif and G-quadruplex, respectively. Moreover, L and R contain a rhodamine green fluorophore (RG) at the border of the gap sequence, while M contains two black hole quencher (BHQ1) dyes at the gap borders. In the hybridized, pseudocatenated state RG and BHQ1 arrange opposite to each other, resulting in FQ (Fig. 1, Supplementary Fig. 1). On addition of ROs that are complementary to the entire gap regions in M, all nanocircles dehybridize from the stalled pseudocatenated into the mobile catenated state. This change in mobility can be visualized by native polyacrylamide gel electrophoresis and is indicated by the appearance of a slower band accompanied by the disappearance of the [3]pseudocatenane band (Fig. 2c, lane 2 versus lane 1). We observed complete conversion of the band corresponding to the [3]pseudocatenane into the [3]catenane. The shift in the electrophoretic mobility of the pseudocatenane/catenane structures most likely originates from the marked difference in their structures. In the pseudocatenane, three circles are locked immobile in an almost linear arrangement, as verified by high-resolution AFM (Fig. 3a, Supplementary Fig. 2). Figure 3a also shows that the head-groups point into opposite directions in almost all observed structures, as was intended by our design.

**Figure 4:**
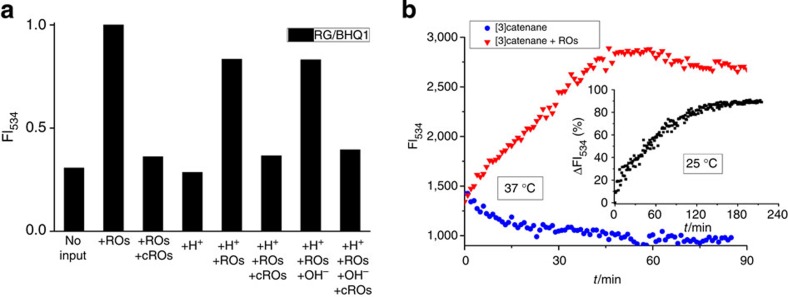
Fluorescence monitoring of the structural changes of DNA nanoarchitectures. (**a**) Fluorescence signal of RG/BHQ1 FQ system in response to the structural changes. The toehold strand displacement is reflected by an increase in the RG fluorescence, while a decrease indicates the reverse process. (**b**) Kinetic data for the toehold-mediated release experiment in the working solution at different temperatures. The addition of ROs induces a sharp increase in the fluorescence in about 1 h at 37 °C, while in about 3 h at 25 °C (inset).

In contrast, circles R and L of the [3]catenane can move unhindered along the central circle M, and can also rotate. By AFM, a shift from the regularly linear into a more compact arrangement can be visualized (Fig. 3b, Supplementary Fig. 3). Interestingly, in agarose gel electrophoresis (Fig. 2b) the electrophoretic mobility is exactly the opposite: now the pseudocatenane migrates slower than the catenane (lane 2 versus lane 1), similar to our previous observations using dsDNA rotaxanes^26^,^28^,^29^. The pseudocatenane→catenane conversion can be reset almost quantitatively by adding oligodeoxynucleotides (ODNs) that are complementary to the ROs (cROs) to remove the ROs (Fig. 2c, lane 3). Further proof for the pseudocatenane→catenane conversion was obtained from FQ data (Fig. 4a). The addition of ROs results in a sharp increase of the fluorescence signal, indicating the separation of RG from the quencher. The fluorescence decreases on addition of the cROs to remove the ROs, and thus the interlocked structure is reset to the original state. The addition of ROs in the presence of H^+^ does not result in an obvious change in the fluorescence signal as compared with the addition of ROs alone. Consequently, the addition of alkali to the sample containing ROs+H^+^ has almost no influence on the fluorescence.

When the pH is lowered to 5, the [3]pseudocatenane undergoes different structural changes (Fig. 2d), but only in the presence of the ROs. Without ROs, the [3]pseudocatenane remains largely unchanged (lane 1), whereas the addition of ROs leads to the disappearance of the [3]pseudocatenane band (lane 2) and the appearance of two new bands. The weaker of the two corresponds to the [3]catenane, whereas the major band that exhibits slightly slower electrophoretic mobility originates from the formation of a bimolecular i-motif by the C-rich branches (C_6_TC_6_) in the two heads of the [3]catenane^37^. This bimolecular i-motif can only form when nanocircles R and L are released from their hybridization with the gaps of M. Importantly, the bimolecular i-motif does not form between two different [3]pseudocatenane molecules, evidenced by the AFM image in Fig. 3c and Supplementary Fig. 4 where dimers of the [3]pseudocatenane are not found. Our previous work^37^ demonstrated that the i-motif-induced interstructural assembly does not occur at submicromolar concentrations but can only be observed at higher concentrations. In contrast, the formation of the bimolecular i-motif in the same [3]pseudocatenane molecule is an intrastructural assembly independent of DNA concentration, and becomes dominant when working at 100 nM concentrations, as we did here. The same is also true for a bimolecular G-quadruplex, which depends on the formation of this bimolecular i-motif in the [3]pseudocatenane structure (vide infra).

The corresponding AFM images are precisely in accordance with this structural conversion under these conditions: The two heads of [3]catenane are closely connected, transforming the [3]catenane into a condensed DNA nanostructure resembling a tetracyclic architecture (Fig. 3c, Supplementary Fig. 4). Similar H^+^-induced structural interconversions were previously observed in other DNA architectures^37^. On addition of cROs, the [3]catenane and in part also the i-motif-condensed [3]catenane convert back into the [3]pseudocatenane (Fig. 2d, lane 3) However, a major part of the i-motif-condensed [3]catenane still remains, suggesting that the highly stable, slowly unfolding i-motif^37^ prevents the [3]catenane from being reset into the [3]pseudocatenane. When the i-motif structure is disrupted by resetting the pH to 8.0 before adding the cROs, the i-motif-condensed [3]catenane quantitatively converts into the [3]catenane (Fig. 2e, lane 1). After adding the cROs to the [3]catenane, almost complete conversion to the [3]pseudocatenane is observed (Fig. 2e, lane 2). These observations clearly demonstrate the programmable assembly of the condensed [3]catenane, induced by ROs and H^+^, and the reset into the [3]pseudocatenane by cROs and OH^−^.

### Reversible logic circuit assembled on the [3]pseudocatenane

**Figure 5:**
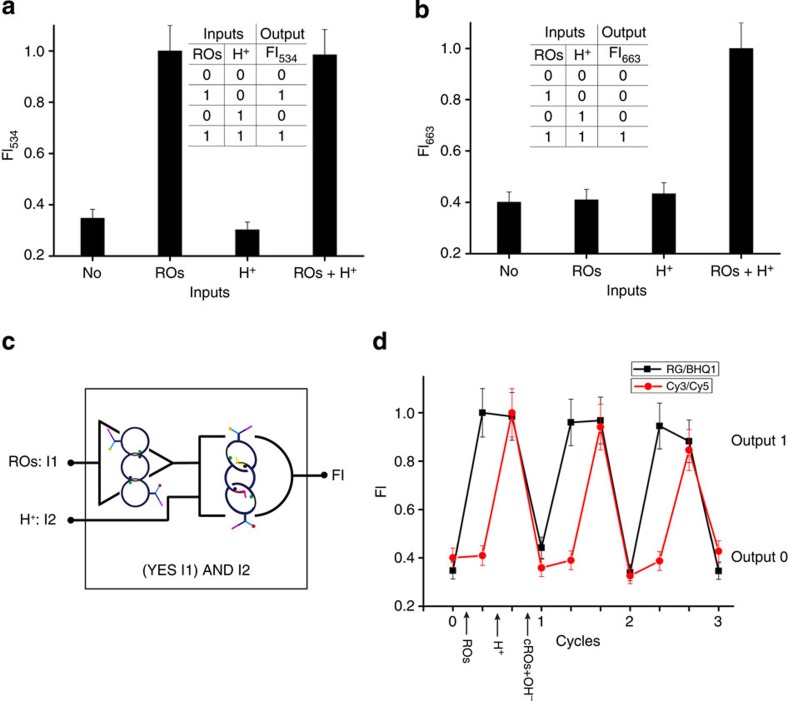
Logic behaviors of dye-labeled [3]pseudocatenane at four input modes. Fluorescence intensity of (**a**) RG-BHQ1 FQ system and (**b**) Cy3-Cy5 FRET system was recorded at 534 nm (FI_534_) and 663 nm (FI_663_), respectively. The normalized FI_534_ and FI_663_ serve as outputs (1/0) with a threshold of 0.6, consistent with two-input ID and AND logic gates, respectively. Insets: corresponding truth tables (corresponding fluorescence spectra: Supplementary Fig. 5). (**c**) Logic circuit that consists of the two tandem gates. Here a one-input YES gate equivalent to the two-input ID gate is adopted. (**d**) Reversibility of the logic circuit in several working cycles measured by fluorescence.

Based on the [3]pseudocatenane architecture and its structural rearrangements, we next employed it to build a reversible logic circuit. We used the fluorescent dye and quencher pairs located at the interlocked gaps and the C-rich branches of L and R to transduce the structural changes triggered by the ROs and pH changes into Boolean operations. The pseudocatenane-to-catenane conversion induced by the ROs is indicated by the change of the RG fluorescence intensity at 534 nm (FI_534_, Fig. 5a, Supplementary Fig. 5a,b). Without any input, RG is located in proximity to BHQ1 in the interlocked structure, which quenches its fluorescence. On input of the ROs, the circles L and R become freely movable in the interlocked structure leading to spatial separation of RG from BHQ1, accompanied by a sharp increase in the fluorescence intensity. The kinetic data for this toehold strand displacement process are shown in Fig. 4b. Without ROs, no increase in the fluorescence is observed, indicating that the [3]pseudocatenane structure is stable under the working conditions at 37 °C. The addition of ROs induces a sharp increase in the fluorescence, demonstrating a structural conversion from the [3]pseudocatenane to the [3]catenane. This comparison confirms that the switch results from the toehold strand displacement rather than from a re-annealing process at 37 °C. A lower temperature needs a longer equilibrium time (Fig. 4b, inset).

Since RG is pH-insensitive in the range of pH 4−9 (refs 4, 38, 39), lowering the pH to 5 only marginally influences the fluorescence spectra (Supplementary Fig. 5a,b). With a threshold of 0.6 for the logic output (1/0), the interlocked architecture behaves as a two-input ID logic gate^40^,^41^ that always identifies with the logic input of ROs. Figure 5a shows the corresponding truth table. The gate is equivalent to a YES gate^42^ with ROs as the input, since the logic output is independent of another input H^+^. The YES gate is controlled by the stalled (output 0) and mobile (output 1) states of L and R.

To indicate the i-motif-induced cyclization of two head-motifs in the interlocked architecture by FRET, two pH-insensitive fluorescent dyes^35^, Cy3 and Cy5, were tethered to the C-rich branch of L and R, respectively. Without ROs, L and R exist in the immobile state and hold the two dyes separate, preventing sufficient FRET to occur. Although the input of the ROs alone allows L and R to move freely, their head-motifs are not held close enough to enable FRET between Cy3 and Cy5. Only when both ROs and H^+^ were employed as inputs, a FRET signal was observed at 663 nm (Fig. 5b, Supplementary Fig. 5c,d) because in this state both dyes are in close enough proximity to enable FRET. Further confirmation of the i-motif induction was achieved by AFM (Fig. 3c). Thus, the system behaves as a two-input logic AND gate^42^, with a threshold of 0.6 for the logic output (1/0). Figure 5b shows the related truth table.

Our observations demonstrate that the AND gate functions on the basis of the mobility of L and R, which is another form of the output of a YES gate. Therefore, the YES gate output in fact serves as one input of the AND gate. These two tandem gates constitute a logic circuit (Fig. 5c), of which the truth table is the same as that of the AND gate (Fig. 5b, inset). Unlike most of the DNA-based logic operations reported previously^19^,^20^,^43^, this DNA logic circuit can be switched repeatedly (Fig. 5d), as a result of the structural changes directed by the interlocked architecture^28^,^35^,^36^.

### A switched DNAzyme under the control of a logic circuit

**Figure 6:**
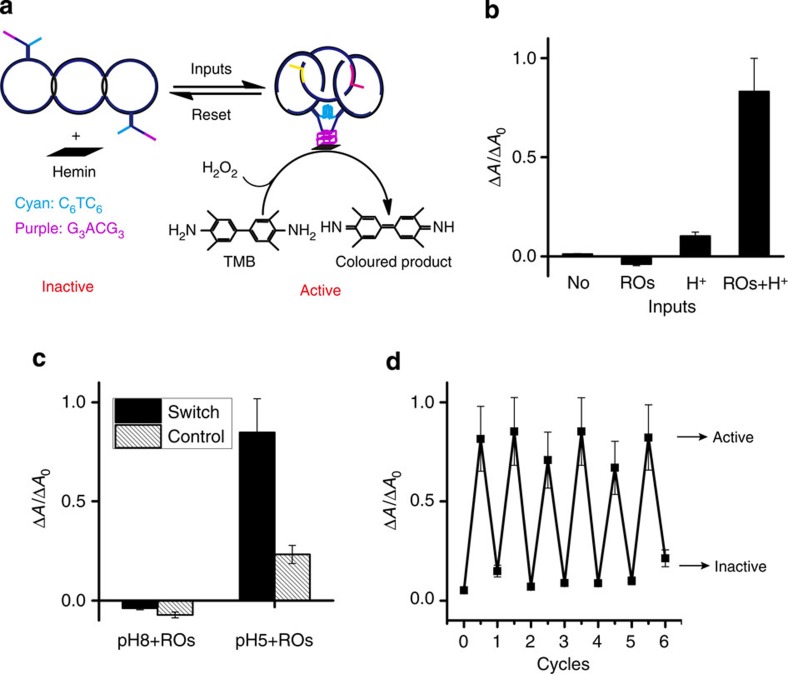
Logically switching a G-quadruplex DNAzyme. (**a**) Working principle of a DNAzyme nanoswitch built on the difference in robustness between the bimolecular i-motif (cyan) and the G-quadruplex (purple) structures on the head-motifs of the interlocked architecture. (**b**) Normalized absorbance of the DNAzyme switch on different inputs, consistent with the output of logic circuit. (**c**) Control experiment for the switch. The control structure is a [3]pseudocatenane with only G-quadruplex structure (that is, no i-motif) on the heads. (**d**) Working cycles of the nanoswitch between the inactive and active states in response to external stimuli.

We next sought to employ the devised reversible logic circuit in a proof-of-concept experiment to control the formation of a bimolecular G-quartet on the interlocked architecture (Fig. 6a). Two tandem G-tracts (G_3_ACG_3_) are included in a branch of the head-motifs. These G-tracts can be directed by base pairing to fold into a bimolecular G-quadruplex in the presence of K^+^ (ref. 44). With hemin as the catalytic cofactor, the bimolecular G-quadruplex exhibits a moderate peroxidase-like DNAzyme activity. However, this G-quadruplex is not robust enough to form a dimer on its own terms that can link the L and R together (Supplementary Fig. 6). Instead, the G-quartet dimer can only form if preceded by the formation of the i-motif at pH 5, consistent with previous observations^37^. This difference in robustness between the bimolecular i-motif and G-quartet is responsible for a pH-dependent DNAzyme activity of the nanocircles (Supplementary Fig. 6). Thus, forming the DNAzyme requires the assistance of i-motif-formation at low pH. This dependence provides a working principle for the logic circuit-tuned DNAzyme nanoswitch on the interlocked architecture.

Note that different peroxidase substrates should be chosen for the G-quartet DNAzyme at pH 8/5. In general, the peroxidase-like DNAzymes perform best when using 2,2′-azino-bis-(3-ethylbenzothiazoline-6-sulphonic acid) (ABTS) as the substrate in alkaline solution, whereas under acidic condition the substrate 3,3′,5,5′-tetramethylbenzidine (TMB) should be used^45^. To directly compare the DNAzyme activity at four input modes of the logic circuit (Fig. 6b), the absorbance of the switch (Δ*A*) is normalized according to that of the individual DNAzyme (Δ*A*_0_) under the same conditions. In the presence of K^+^, the DNAzyme displays a moderate peroxidase activity on input of both ROs and H^+^, whereas it is inactive in the other three cases. Apparently, the performance of the DNAzyme depends on the logic behaviours of the YES–AND circuit (Fig. 5), and is therefore ‘tuned’ by the logic circuit. In contrast to the switch (Fig. 6c, black bars), a control interlocked architecture that lacks the i-motif displays almost no DNAzyme activity, regardless of inputs (Fig. 6c, grey bars). This result again indicates that the bimolecular G-quadruplex alone is unable to connect the head-motifs of the interlocked architecture, consistent with the observations for the non-interlocked DNA circles (Supplementary Fig. 6). This difference in switching behaviour serves as the basis for operating the logic circuit, and allows the DNAzyme switch to function repeatedly and fully reversibly (Fig. 6d)^4^,^8^,^37^,^38^,^39^,^46^,^47^.

## Discussion

We have demonstrated the programmable assembly of [3]pseudocatenane with two branched-out heads functionalized with i-motif and G-quadruplex DNAs. The mobility of interlocked circles triggered by the ROs, and the proton-induced cyclizations of their head-motifs are verified by gel electrophoresis, AFM and fluorescence experiments. The whole process can be reset by the addition of cROs and an increase of the pH to 8, to remove the ROs and to disrupt the i-motif structure, respectively. The mobility and cyclization steps can easily be monitored by the RG/BHQ1 and Cy3/Cy5 system, respectively, that behave as two tandem logic gates (YES and AND). We show that the interlocking allows for the direct coupling between the i-motif assembly and the formation of the G-quadruplex structure. By this means, a novel reversible fluorescent logic circuit is built and then employed to control a repeatedly operating DNAzyme nanoswitch on the basis of difference in robustness between the designed bimolecular i-motif and the G-quadruplex.

An important feature of the here described logic device is its robust reversibility. To our knowledge, so far only a single example of a reversible DNA-based logic gate has been described by Turberfield and colleagues^21^. They described a reversible AND gate comprising a DNA hairpin motif that can balance between ON and OFF conformations depending on toehold exchange of complementary input ODNs. The ON-state was reported by an increase in fluorescence by an opened molecular beacon. As pointed out by Turberfield^21^, reversibility of DNA logic circuits is important, (i) to better respond to changes in inputs, (ii) to reduce, or even avoid, error accumulation, and (iii) to prevent hysteresis resulting from sequential inputs. We introduce a completely different system that consists of tandem YES and AND gates, and show how to employ a reversible DNA logic circuit to control a complex DNA nanostructure by switching DNAzyme activity reversibly and repeatedly. These systems can be envisioned to be combinable with other reversible DNA logic circuits to achieve further cascading of logic gates. The two heterogeneous inputs, H^+^ and toehold ROs, could potentially be coupled to more complex future functional DNA nanostructures. To achieve a possible future coupling of our circuit with other DNA computing devices in follow-up applications, an option would be to operate our system via photoswitched ROs^28^ and light-controlled pH change^48^,^49^ that has successfully been applied to non-reversible DNA logic arrays^50^. Importantly, no additional ROs, cROs and proton are required for our reversible DNA circuit, which should further facilitate the use of this system as a component in larger DNA computing circuits.

Our study demonstrates how to harness unique and precisely controllable structural features of interlocked DNA nanoarchitectures for functionalization, and provides a further important step towards integrating DNA computing with functional nanodevices. Such reversible logic circuits integrated into functional nanodevices may serve as a guide for intelligent DNA nanorobots to manipulate cascade reactions in biological systems^14^,^51^,^52^.

## Methods

### Synthesis of dsDNA [3]pseudocatenane

To synthesize the dsDNA [3]pseudocatenane, the one-gap flanking circles L and R with a branched-out head-motif were synthesized as described before^37^ and purified by weak anion exchange high-performance liquid chromatography (HPLC)^26^ (Sequences: Supplementary Table 1). Then, the 1:1 mixture of L and R was incubated at 15 °C overnight with three hybridized component sequences constituting the circle M to accomplish the threading. Finally, the last sequence of M was added together with T4 DNA ligase (6 μl, 30 U) and the ligation buffer (40 mM Tris–HCl, 10 mM MgCl_2_, 10 mM DTT, 0.5 mM ATP, pH 7.8). The ligation was carried out at 15 °C overnight to produce the stable pseudocatenane structure, followed by HPLC purification. The final product was kept in the DNA stock buffer (pH 8, 40 mM Tris–AcOH buffer, 50 mM NaCl).

### Fluorescent logic circuit

The dye-labeled [3]pseudocatenane was synthesized, highly purified by weak anion exchange HPLC, and prepared in the DNA stock buffer plus 2 mM Mg(OAc)_2_ at a working concentration of 100 nM. Under this condition, the interlocked duplex sections of [3]pseudocatenane are quite stable (T_m_~43 °C), allowing it to work at biological temperature (37 °C). Briefly, the [3]pseudocatenane solution was incubated with 10 equivalents of RO-L and RO-R (that is, ROs) at 37 °C for 5 h to accomplish the pseudocatenane-to-catenane structural conversion. The sample solution was kept at 4 °C after slowly cooling to 4 °C. Then, 1.2% (v/v) of 2 M HCl was added to adjust pH to 5, allowing the i-motif DNA to fold properly at 4 °C overnight to achieve the cyclization of the heads in the interlocked architecture. The structural changes were monitored by FQ and FRET. Fluorescence signals were recorded using an EnSpire Multimode Plate Reader (PerkinElmer).

To work repeatedly, 20 equivalents of cROs together with 1.2% (v/v) of 2 M NaOH were added into the above solution to reset the system back to the original state. In the following circles, 20 equivalents of ROs and cROs are alternatively added to the solution, and performed as described above. Here ‘equivalent’ always refers to the initial concentration (100 nM) of [3]pseudocatenane.

### Gel electrophoresis

The assembled interlocked architectures (raw product) were analysed by non-denaturing polyacrylamide gel (6%) prepared in 40 mM, pH 8/5 Tris–AcOH buffer. Gel electrophoresis was performed in the same buffer at 4 °C for 6 h at 6 V cm^−1^, pH 8, or 8 V cm^−1^, pH 5. The gel was then stained with GelRed, and photographed under ultraviolet light.

A quantity of 2.5% agarose gel was also employed to analyse the assembly of interlocked DNA structures in pH 8, 20 mM Tris–HOAc buffer plus 2 mM MgCl_2_ under 8 V cm^−1^ for 1 h at room temperature. The gel was stained with GelRed and photographed under ultraviolet light.

### AFM

The formed DNA nanoarchitectures (pure product) were confirmed by AFM: Nanowizard 3, JPK Instruments; measuring modes: HyperDrive in liquid and AC in air; substrate: mica with linear polyethylene imine (AC mode) or polyornithine (HyperDrive mode) as adhesive. Just before measurements, the above-prepared sample was diluted to 10 nM with 40 mM Tris–AcOH (pH 8/5) containing 12.5 mM MgCl_2_ and 2.5 mM EDTA.

### Colorimetric measurements

The [3]pseudocatenane-based nanoswitch (raw product) of a final concentration at 100 nM was operated in the presence of 20 mM KCl, with an equivalent of hemin as the catalytic factor. The DNAzyme activity of the nanoswitch was characterized at pH 5 in 0.2 mM TMB mixed with 1 mM H_2_O_2_, whereas at pH 8 1 mM ABTS plus 1 mM H_2_O_2_ was used. The absorbance (*A*) of the coloured products of 120-min TMB and 6-min ABTS oxidation reactions was recorded at 451 and 421 nm, respectively, by a ultraviolet–visible spectrometer (JASCO, V-630). Meanwhile, the absorbance resulting from the catalysis of hemin (*A*_hemin_) and individual G-quadruplex DNAzyme (*A*_0_) was recorded under the same conditions. The variable Δ*A*/Δ*A*_0_=(*A*−*A*_hemin_)/(*A*_0_−*A*_hemin_) was adopted to properly reveal the activity change of the DNAzyme nanoswitch in response to different inputs.

## Author contributions

T.L. and M.F. designed the project; T.L. and F.L. performed the experiments; T.L., F.L. and M.F. wrote the paper.

## Additional information

**How to cite this article**: Li, T. *et al.* Interlocked DNA nanostructures controlled by a reversible logic circuit. *Nat. Commun.* 5:4940 doi: 10.1038/ncomms5940 (2014).

## Supplementary Material

Supplementary Information Supplementary Figures 1-6 and Supplementary Table 1
