# Supplementary material for: Interlocked DNA nanostructures controlled by a reversible logic circuit
Source: Nat Commun. 2014 Sep 17;5:4940. doi: 10.1038/ncomms5940 (PMC4199106; doi:10.1038/ncomms5940)
Supplement: Supplementary Information — Supplementary Figures 1-6 and Supplementary Table 1 [file ncomms5940-s1.pdf]

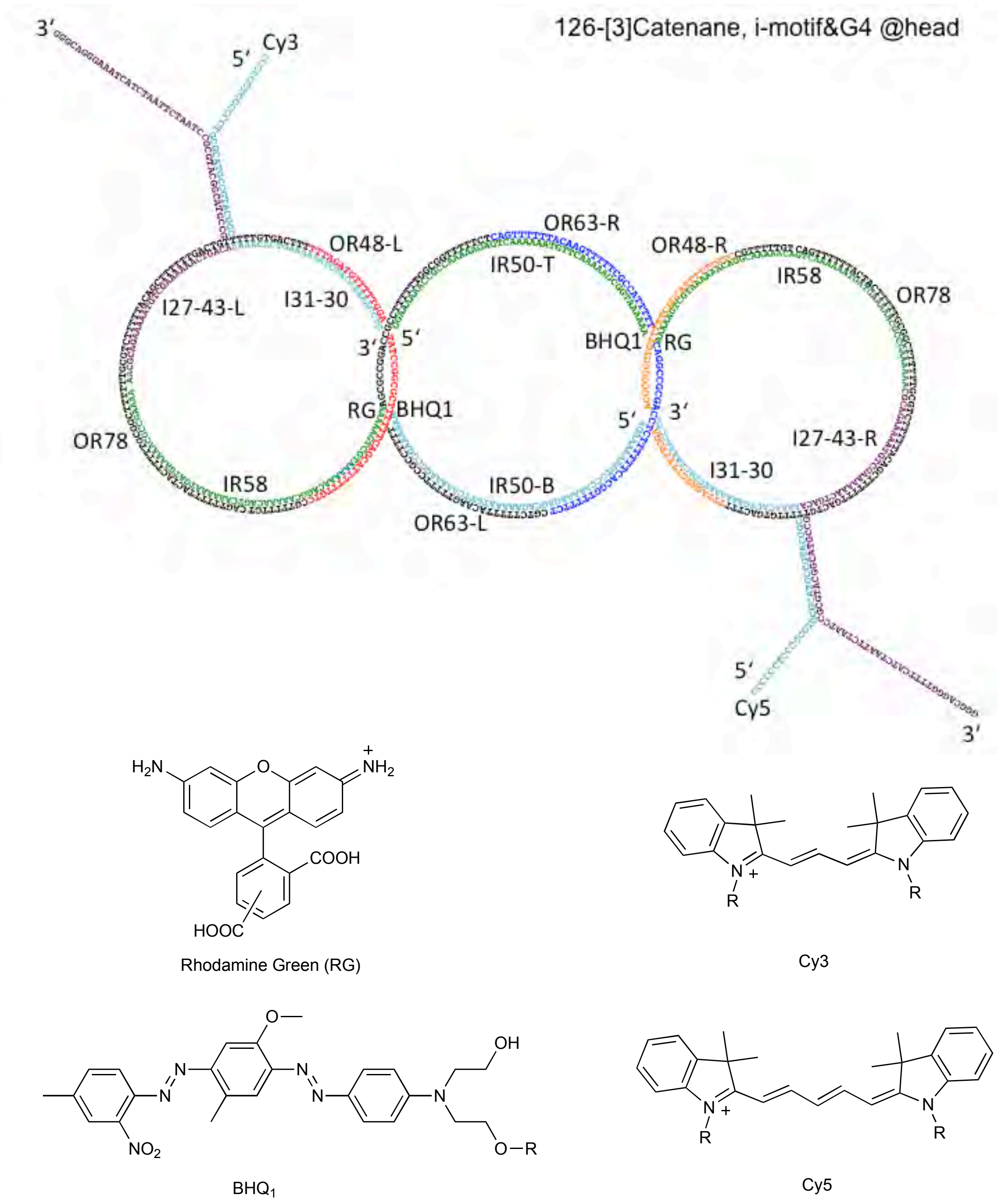

Supplementary Figure 1. Dye-labeled [3]catenane and molecular structures for the used fluophors.

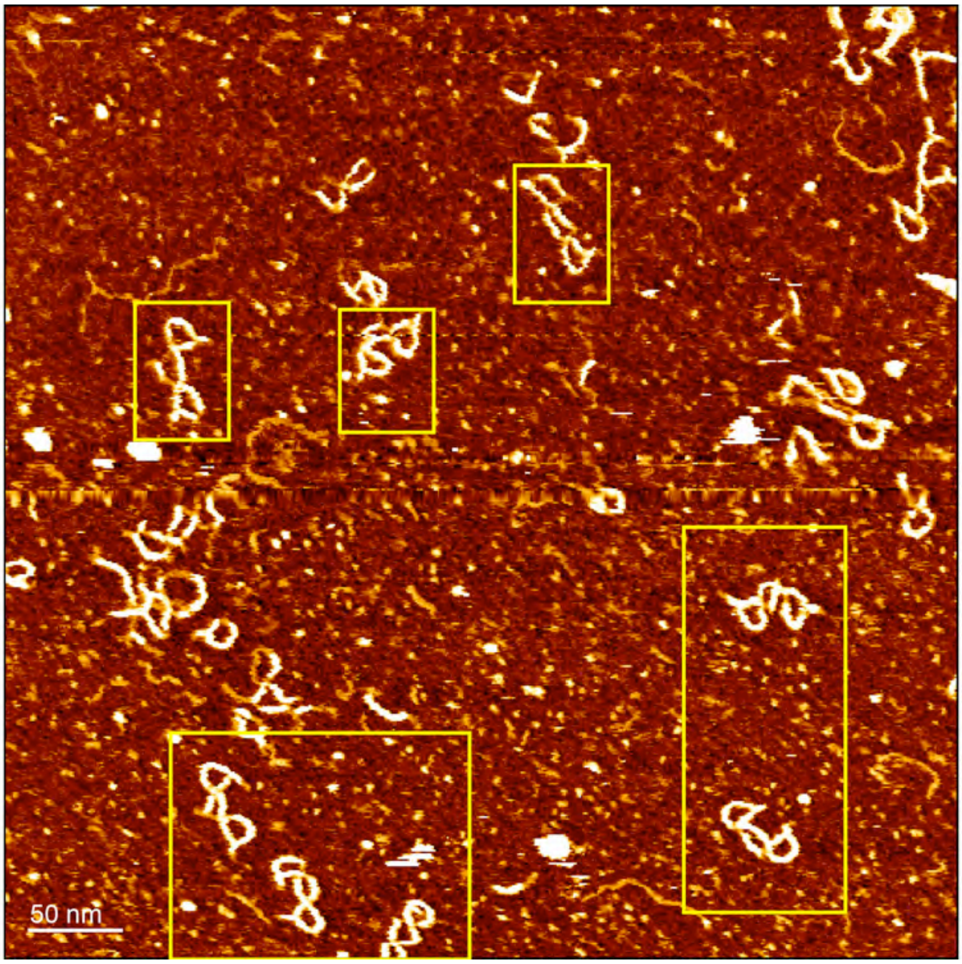

Supplementary Figure 2. Entire AFM images of [3]pseudocatenane sample corresponding to Figure 3a in the text. The cut-outs shown in Figure 3a are marked with yellow frames.

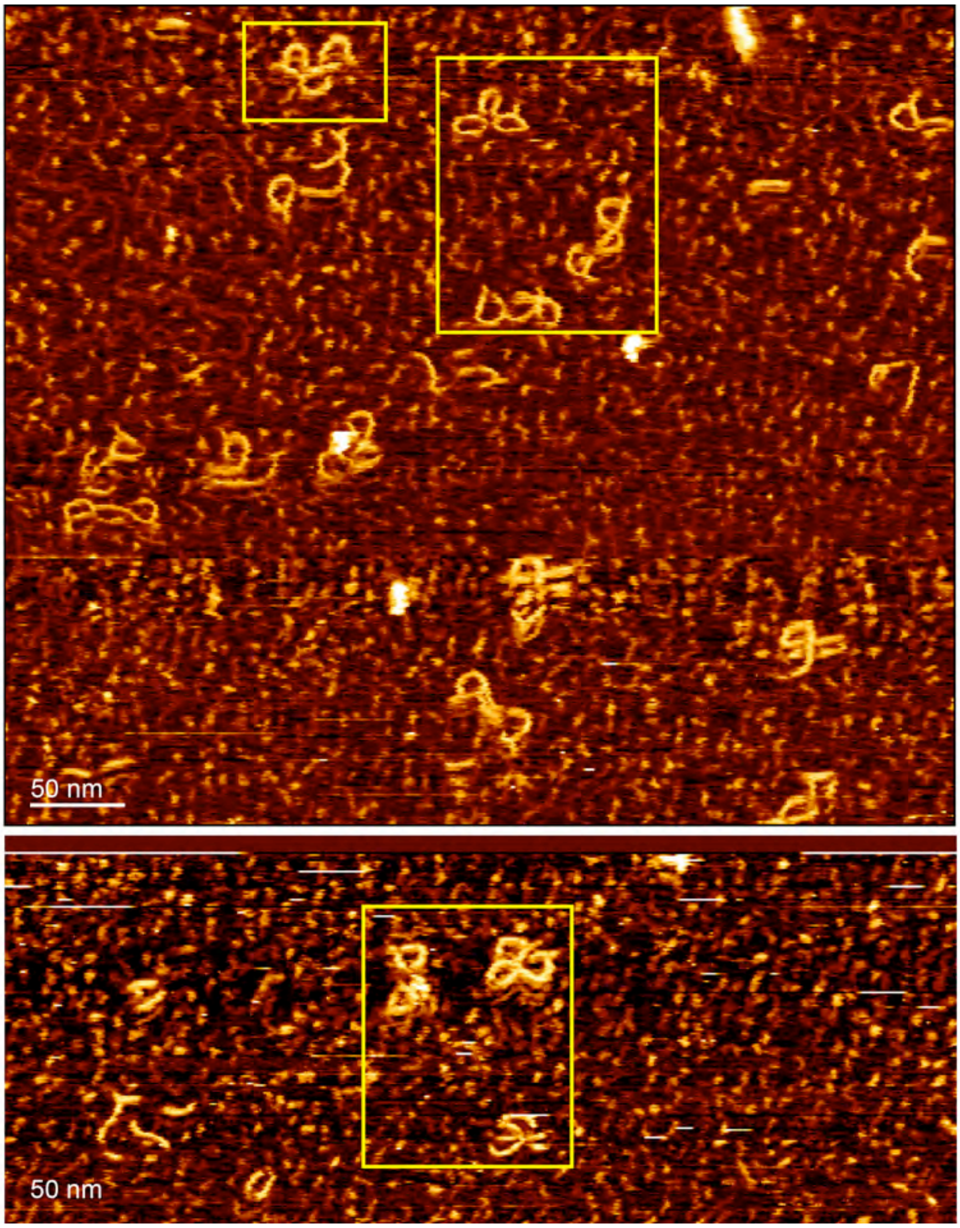

Supplementary Figure 3. Entire AFM images of [3]pseudocatenane sample after addition of ROs corresponding to Figure 3b in the text. The cut-outs shown in Figure 3b are marked with yellow frames.

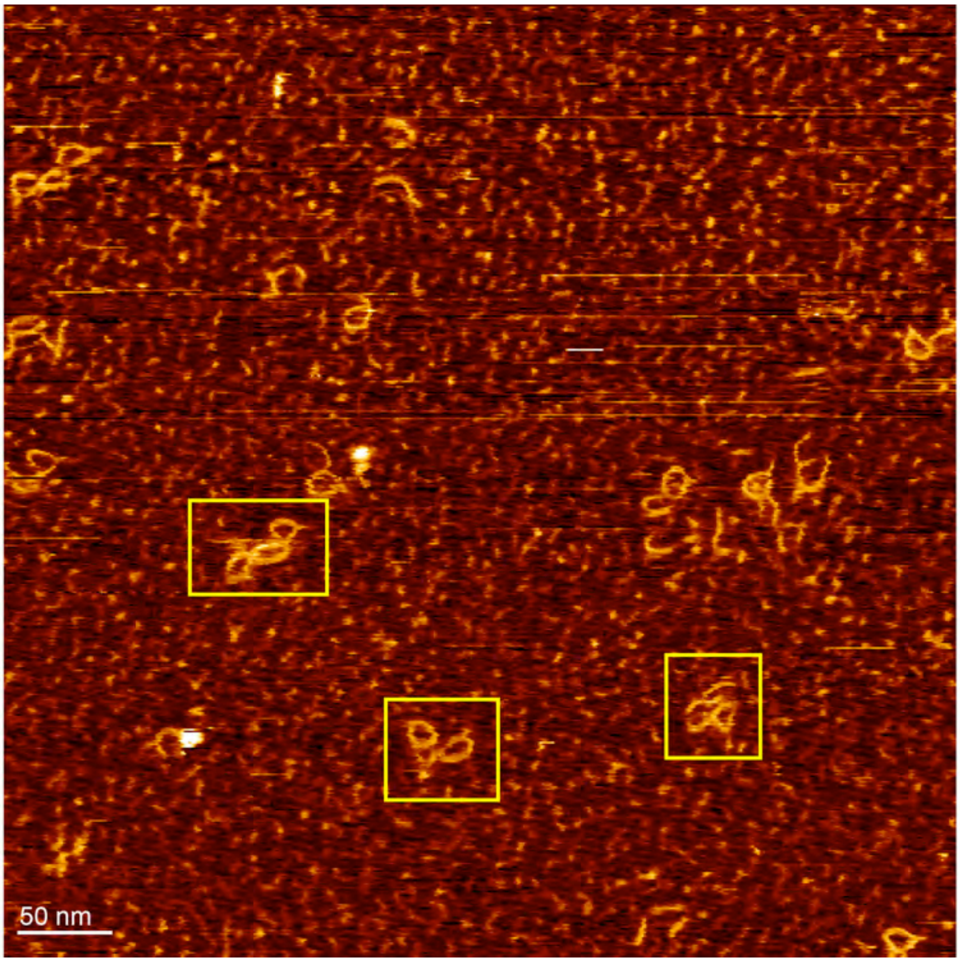

Supplementary Figure 3. continued from last page.

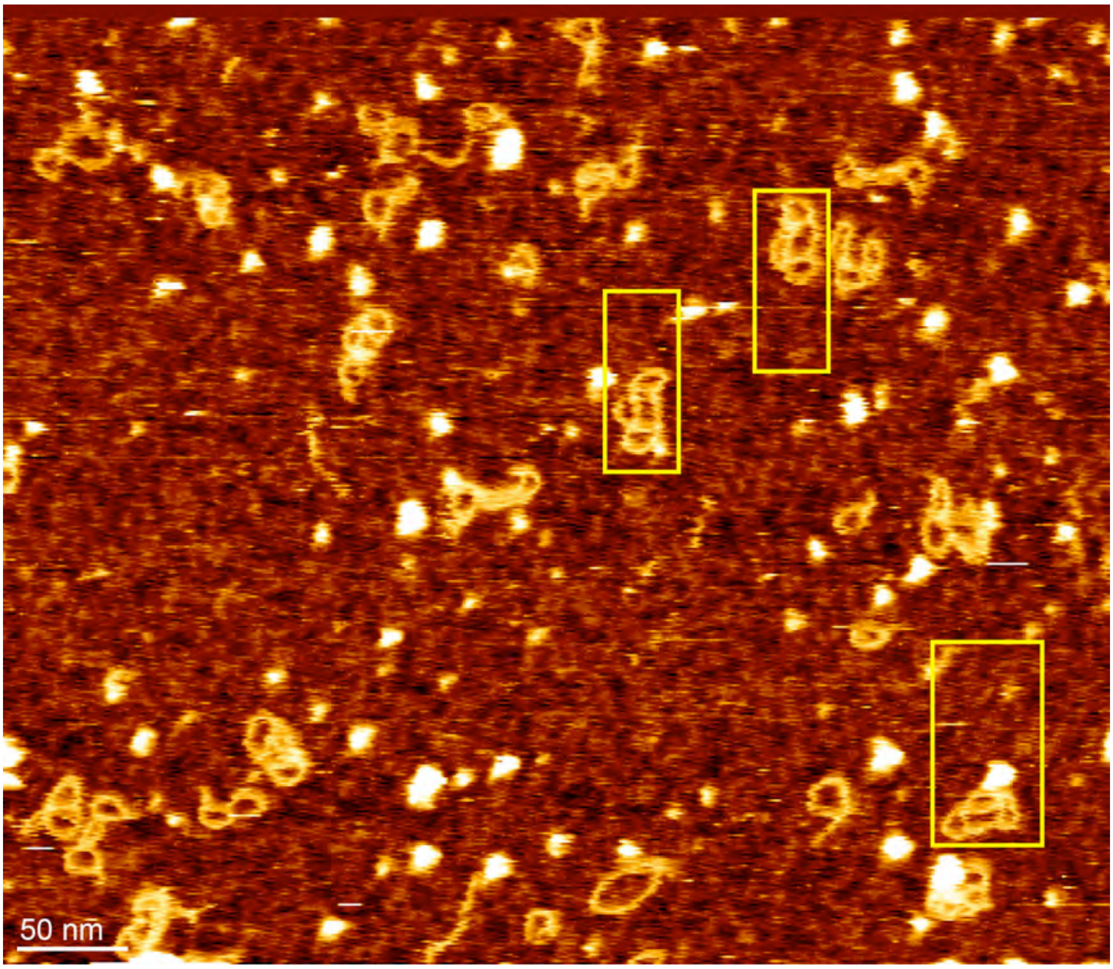

Supplementary Figure 4. Entire AFM images of [3]pseudocatenane sample after addition of ROs and H^+ corresponding to Figure 3c in the text. The cut-outs shown in Figure 3c are marked with yellow frames.

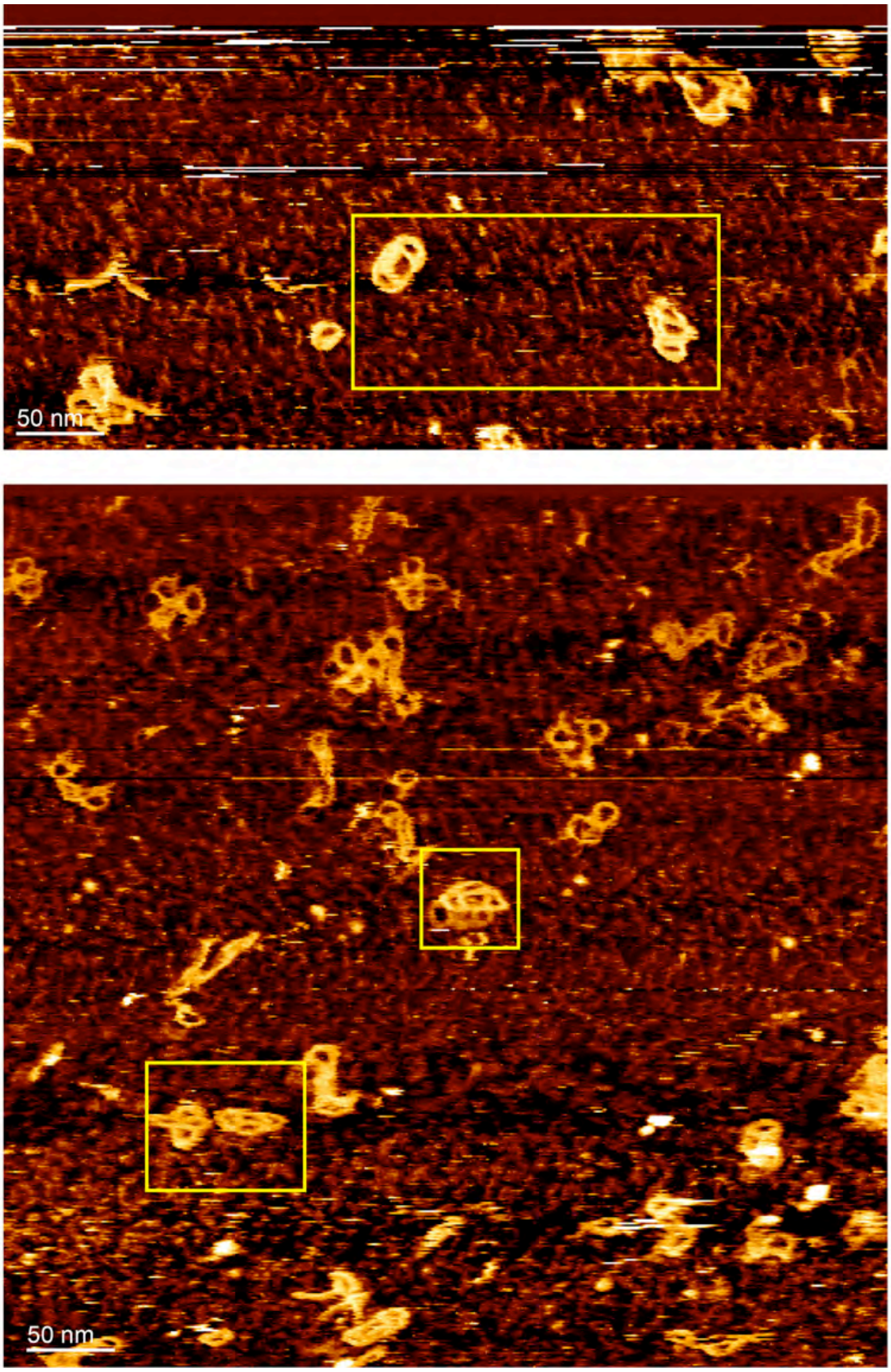

Supplementary Figure 4. continued from last page.

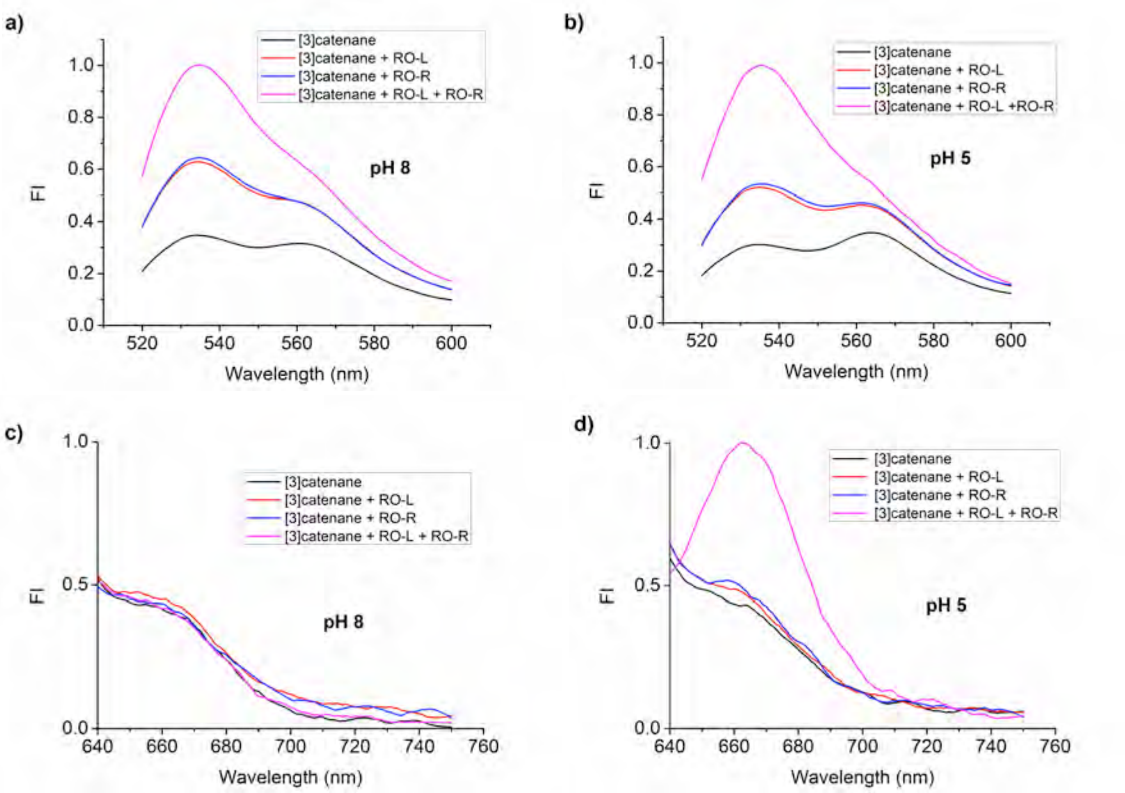

Supplementary Figure 5. Fluorescence spectra of the RG/BHQ1 FQ (a, b) and Cy3/Cy5 (c, d) FRET systems for [3]pseudocatenane in response to different inputs. From them we can conclude that RO-L and RO-R almost have an equal influence on the system, although their sequences are different. For this reason, RO-L and RO-R are regarded as one input (i.e. ROs) and used at 1:1 molar ratio for Boolean operations.

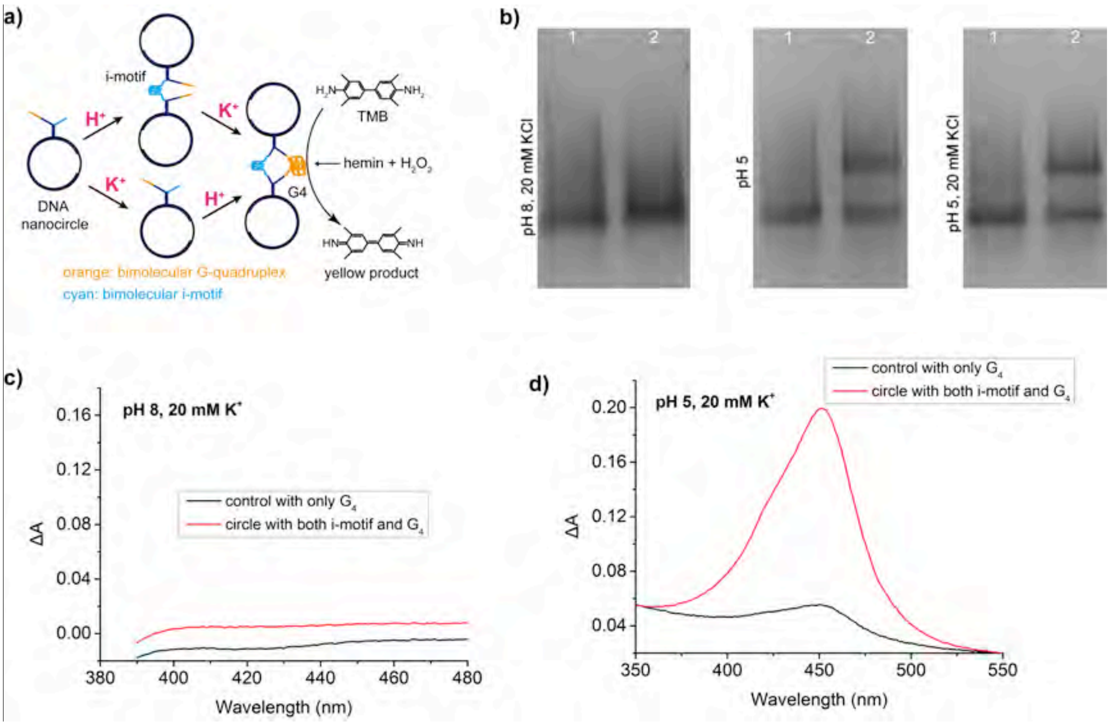

Supplementary Figure 6. a) pH-dependent DNAzyme switch built on the robust difference between the bimolecular i-motif and G-quadruplex structures on the head of DNA circle. **b)** Electrophoretogram of DNA nanocircles in 2.5% agarose gel under different conditions: 1, control circle with only G-rich DNA branch on the head; 2, the circle with C-rich and G-rich DNA branches. The circle dimer is only formed in the presence of proton (middle and right panels), namely, the bimolecular i-motif is robust enough to connect two circles whereas the bimolecular G-quaduplex does not so (left panel). In addition, the existence of G-quadruplex does not promote the formation of circle dimer at all (middle panel vs right panel). **c)** DNAzyme activity of two circles (100 nM) with one equivalent of hemin at pH 8 in 1 mM ABTS mixed with 1 mM H_2O_2 (6-min reaction). **d)** DNAzyme activity characterized at pH 5 in 0.2 mM TMB mixed with 1 mM H_2O_2 (120-min reaction).

Supplementary Table 1. DNA sequences for the synthesis and assembly of [3]catenanes.

**Name**	**DNA Sequences**
OR48-L	Phos-CTGTTTTTTACGACTTTTTTCGCGGCCTATGCAGGTTTTTGTAGATTT
OR48-R	Phos-CTGTTTTTTACGACTTTTTGTCCGGCGCATGCAGGTTTTTGTAGATTT
OR78	Phos-TTTCAGTGTTTTTGTCAGTTTTTTCGACATTTTTCTGCGTTTTTTCGGCGTTTTTCATCATTTTTTGACTGTTTTTGC
RG-IR58	RG-AAAAAGTCGTAAAAAACAGGCAAAAACAGTCAAAAAATGATGAAAAACGCCGAAAAAA
Cy3-I31-30	Cy3-CCCCCCTCCCCCCTGCGCATGCCGTACGGCAAAACACTGAAAAAATCTACAAAAACCTGCA
Cy5-I31-30	Cy5-CCCCCCTCCCCCCTGCGCATGCCGTACGGCAAAACACTGAAAAAATCTACAAAAACCTGCA
Control	TTTCCTTTCCATTTGCGCATGCCGTACGGCAAAACACTGAAAAAATCTACAAAAACCTGCA
I27-43-L	Phos-CGCAGAAAAATGTCGAAAAAACTGACAGCCGTACGGCATGCGCCTAATCTTAATCTACTAAAGGGACGGG
I27-43-R	Phos-CGCAGAAAAATGTCGAAAAAACTGACAGCCGTACGGCATGCGCCTAATCTTAATCTACTTTTGGGACGGG
IR50-T-BHQ1	GGAAAAAACCGCCAAAAAGAGTCAAAAAATGTTCAAAAAGCGGTAAAAAA-BHQ1
IR50-B-BHQ1	AGAAAAAAGTGCCAAAAAGAGCAGAAAAATGTTCAAAAAGGCGGAAAAAA-BHQ1
OR63-L	Phos-TCTTTTTGGCGGTTTTTTCCGCCAGGCCGCGACTTTTTTCCGCCTTTTTGAACATTTTTCTGC
OR63-R	Phos-TCTTTTTGGCACTTTTTTCTCCAGCGCCGGACGTTTTTTACCGCTTTTTGAACATTTTTTGAC
RO-L	GTCGCGGCCTGGCTGATCACT
RO-R	CGTCCGGCGCTGGAGACTTGA
cRO-L	AGTGATCAGCCAGGCCGCGAC
cRO-R	TCAAGTCTCCAGCGCCGGACG
